# Supplementary material for: Characterisation of laminar and vascular spatiotemporal dynamics of CBV and BOLD signals using VASO and ME-GRE at 7T in humans
Source: Imaging Neurosci (Camb). 2024 Aug 13;2:imag-2-00263. doi: 10.1162/imag_a_00263 (PMC12290604; doi:10.1162/imag_a_00263)
Supplement: Supplementary Material [file imag_a_00263-supp.pdf]

Supplementary Materials

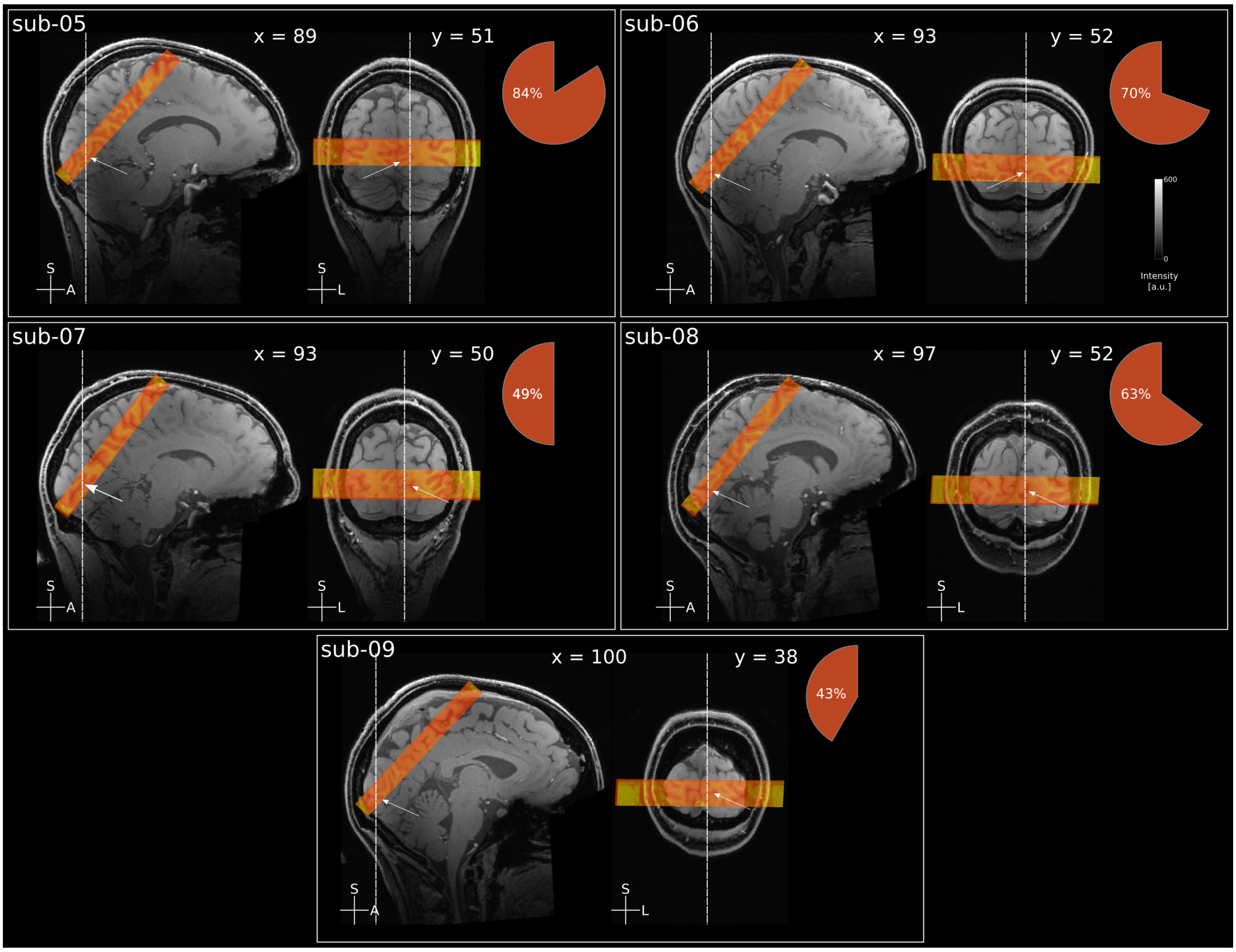

Figure S1: Functional data coverage for all participants. White arrows indicate location of the calcarine sulcus, based on anatomical landmarks. Because of the small FOV and number of slices, it was not always possible to include the entire calcarine sulcus without foldover. For example in participant sub-09, the sulcus folded down from the opening. The pie chart indicates an estimate of the percentage of V1 that was covered for a given participant.

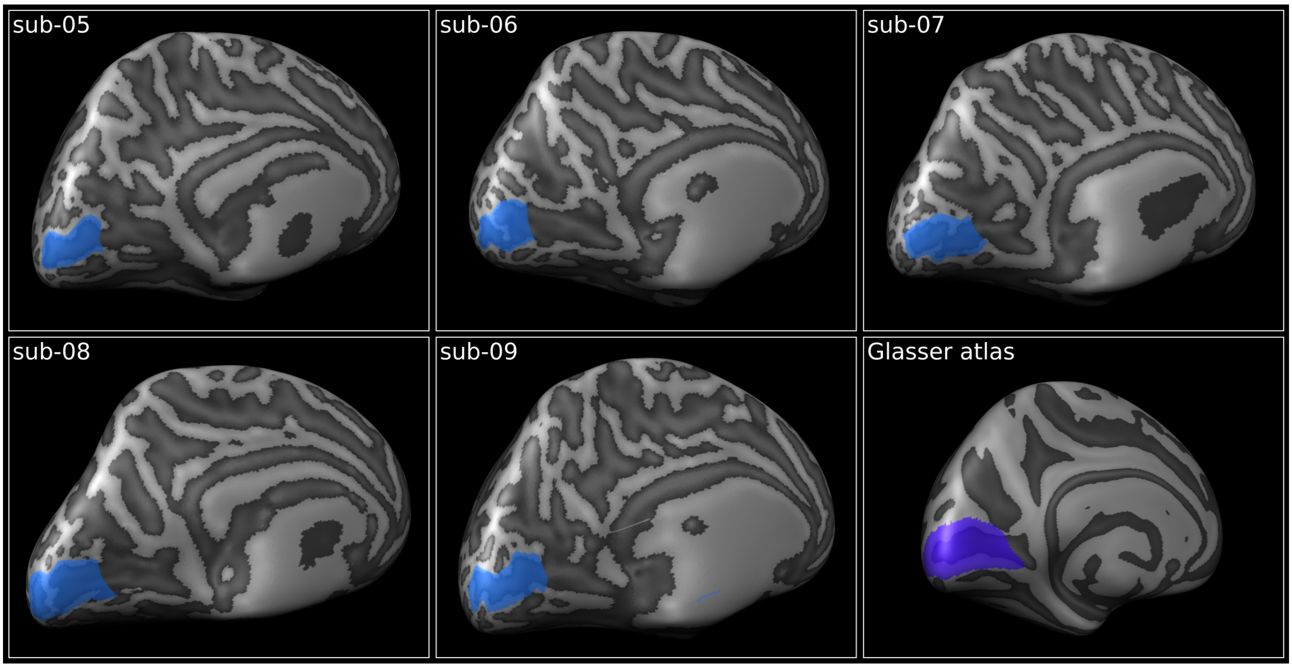

Figure S2: Outline of the posterior calcarine sulcus for all participants. Based on the Glasser atlas, we drew manual outlines of the calcarine sulcus (indicating V1) on the inflated cortical surface for all participants. These patches were used to estimate the overlap between the functional coverage and V1 (see **Supplementary Figure S1**). Furthermore, we used them to visually ensure that our ROIs were located in V1.

Table S1: Session overview of participants.

Participant	Session 1	Session 2	Session 3	Session 4	Session 5	Number func. runs
sub-01-sub-04	Pilot	-	-	-	-	-
sub-05	MP2RAGE 5 short ITI	MP2RAGE ME-GRE	7 short ITI	6 long ITI	6 long ITI	12 short ITI 12 long ITI
sub-06	MP2RAGE 6 short ITI	7 short ITI	6 long ITI	MP2RAGE ME-GRE	6 long ITI	13 short ITI 12 long ITI
sub-07	MP2RAGE 5 short ITI	6 long ITI	6 long ITI	6 long ITI	MP2RAGE ME-GRE	5 short ITI 18 long ITI
sub-08	MP2RAGE 4 long ITI	6 long ITI	5 long ITI	MP2RAGE ME-GRE	6 long ITI	21 long ITI
sub-09	MP2RAGE 5 long ITI	ME-GRE	7 long ITI	6 long ITI	6 long ITI	23 long ITI

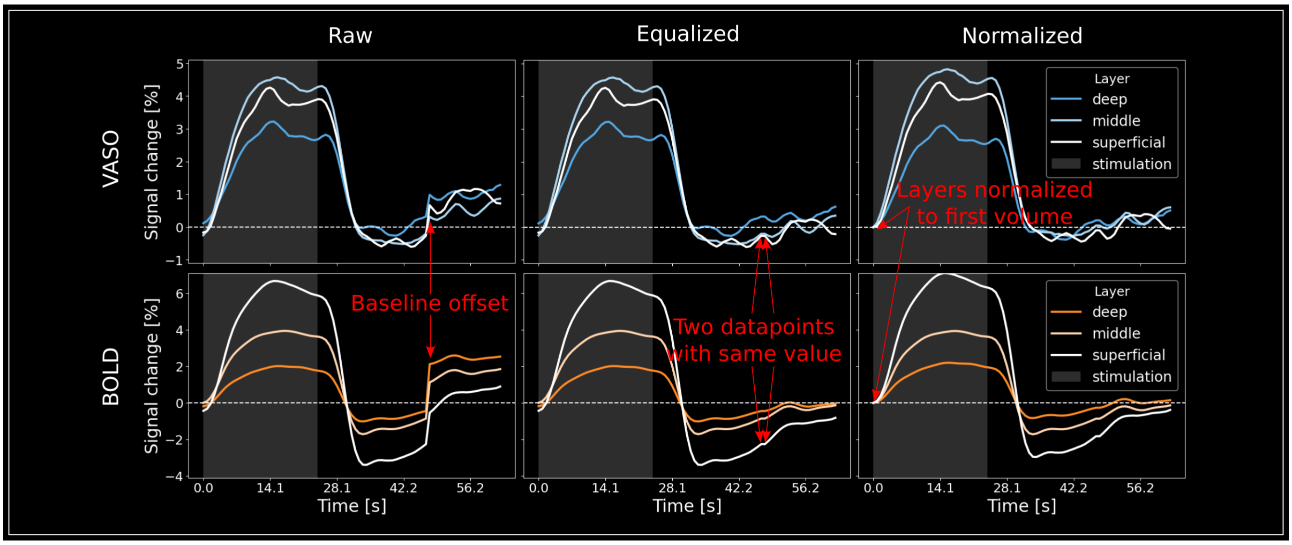

Figure S3: Equalization and normalization procedure. Left: VASO and BOLD ERAs for one individual participant (sub-06) in terms of raw extracted % signal change. A shift in baseline is clearly visible between sessions with short to long ITIs. Middle: Volumes with long ITIs were matched with the last time point of the short ITI sessions. This leads to two timepoints with the same value. Right: Finally, we set the first volume of each layer to 0 and adjusted all remaining volumes accordingly.

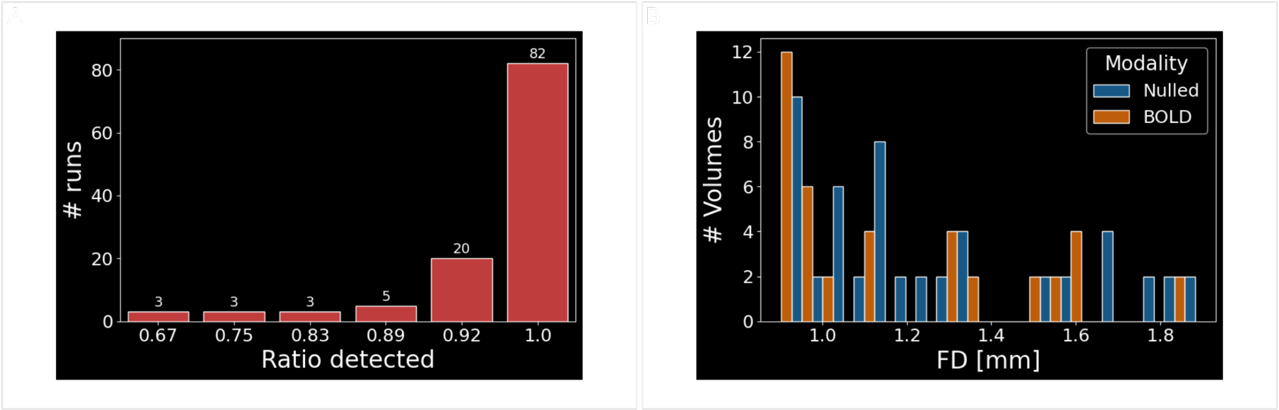

Figure S4: Attention-task performance and motion. A Number of targets detected divided by the total number of targets per run. Performance was high overall, with only a limited number of runs in which multiple targets were undetected. **B** Distribution of volumes with FD > 0.9 mm across BOLD and nulled acquisitions. We acquired a total of 117088 volumes (nulled and BOLD combined). Only 92 volumes (<0.08%) showed framewise displacements (FDs) greater than our voxel size (0.9 mm). 40 (in 16 individual runs) of those were in BOLD and 52 (in 12 individual runs) were in nulled time series and FD never exceeded 1.88 mm. We therefore did not exclude any data based on motion.

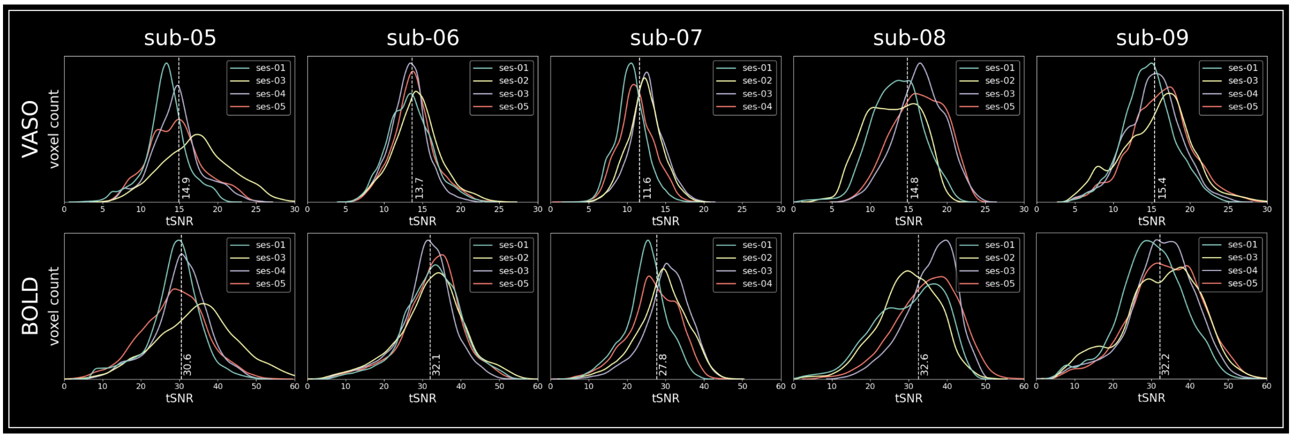

Figure S5: VASO and BOLD tSNR for each participant and session individually. VASO and BOLD tSNR values are mostly uniform across sessions with minor differences between participants. Values were extracted from the same regions of interest as GLM results and signal changes

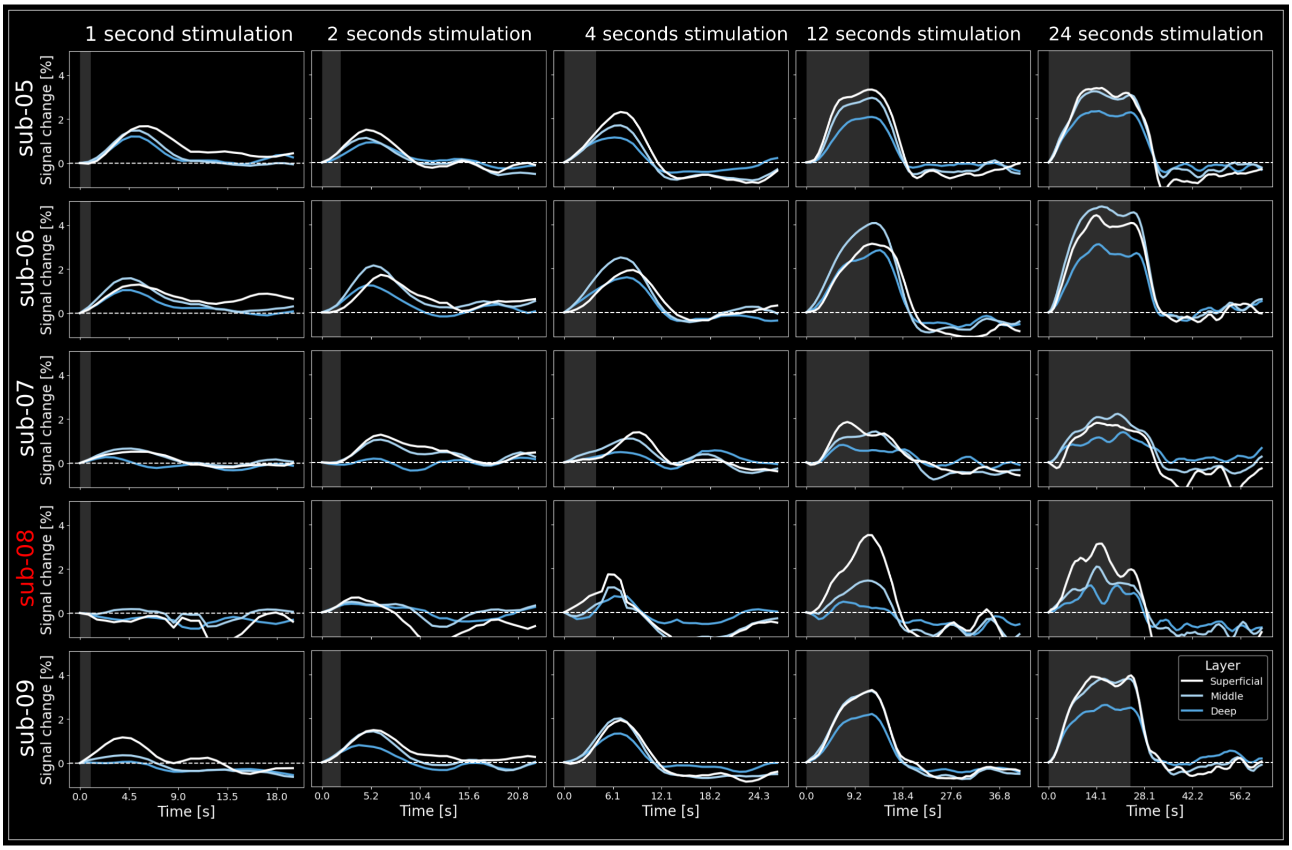

Figure S6: VASO results of individual participants.. Same as **Figure 3B** but for all participants individually. Data averaged across all sessions and normalized to zero to the first datapoint for each layer. Note that participant sub-08 did not show any activation in response to stimulation of 1 second and only weak activation in response to 2 second stimulation. This prohibits some of the normalization procedures conducted in this study (e.g. division by zero for data in **Figure 7**. Therefore, we excluded this participant in the group average plots.

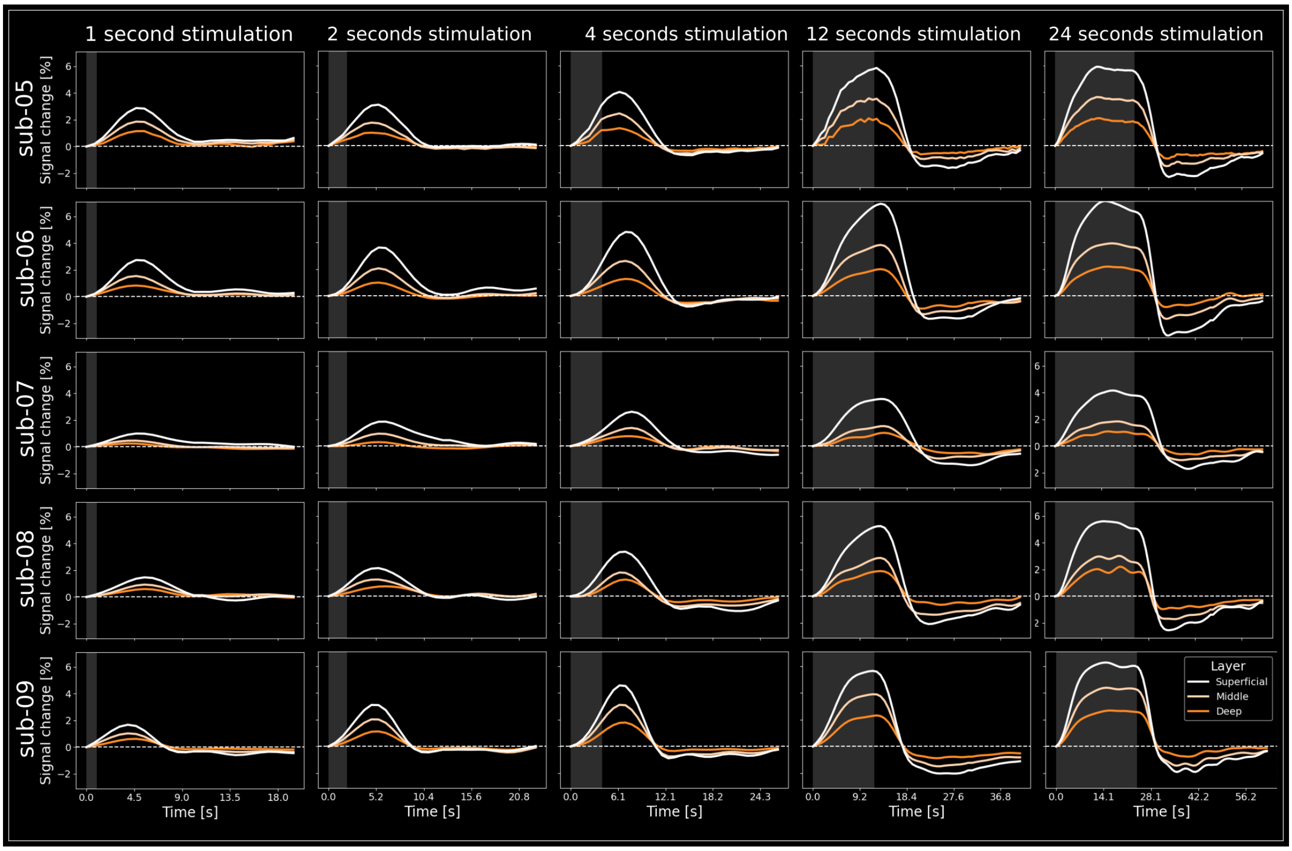

Figure S7: BOLD results of individual participants. BOLD results of individual participants. Same as **Figure 3B** but for all participants individually. Data averaged across all sessions and normalized to zero to the first datapoint for each layer.

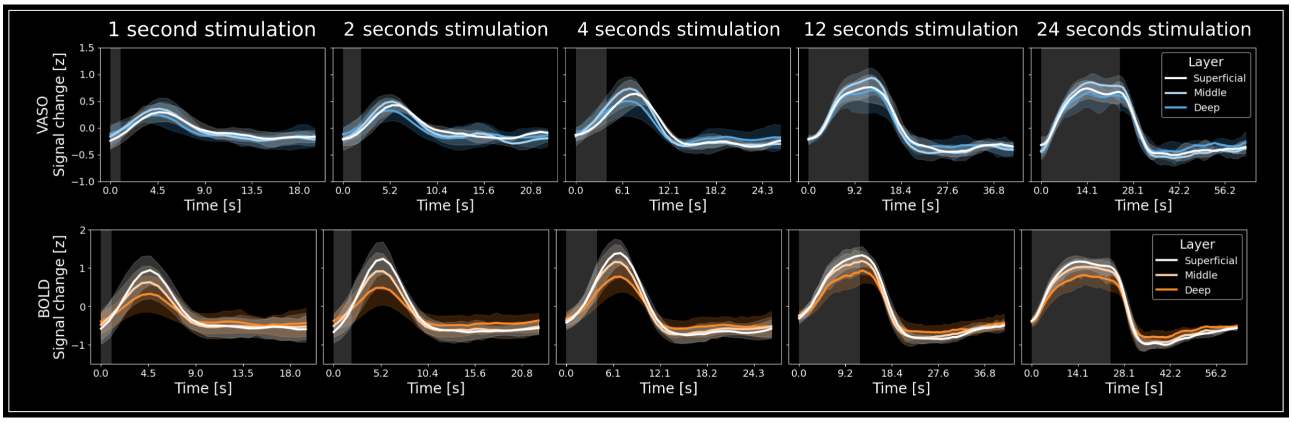

Figure S8: Group-level VASO and BOLD (z-scored). Same as **Figure 3B** but z-scored.

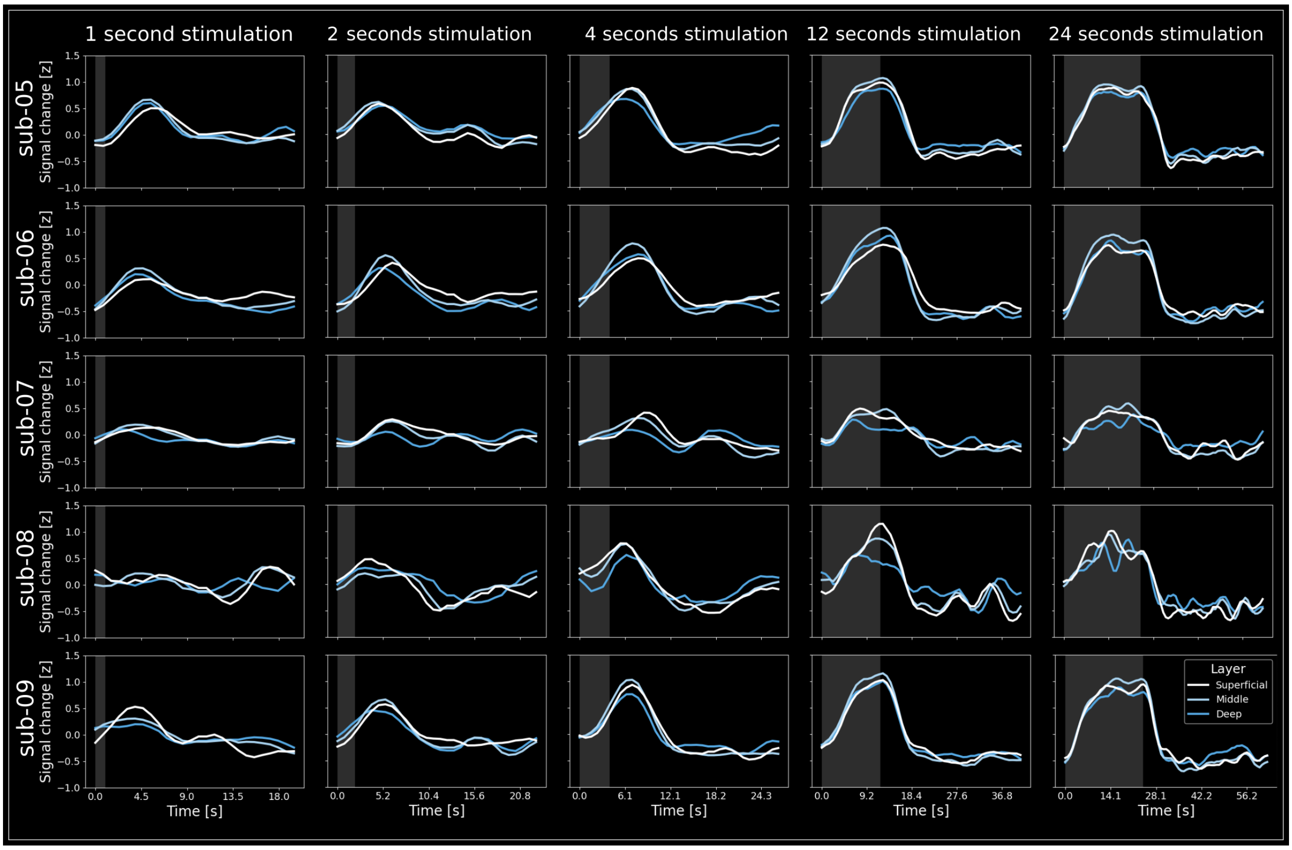

Figure S9: Z-scored VASO results of individual participants. Same as **Figure S6** but with z-scored signal changes.

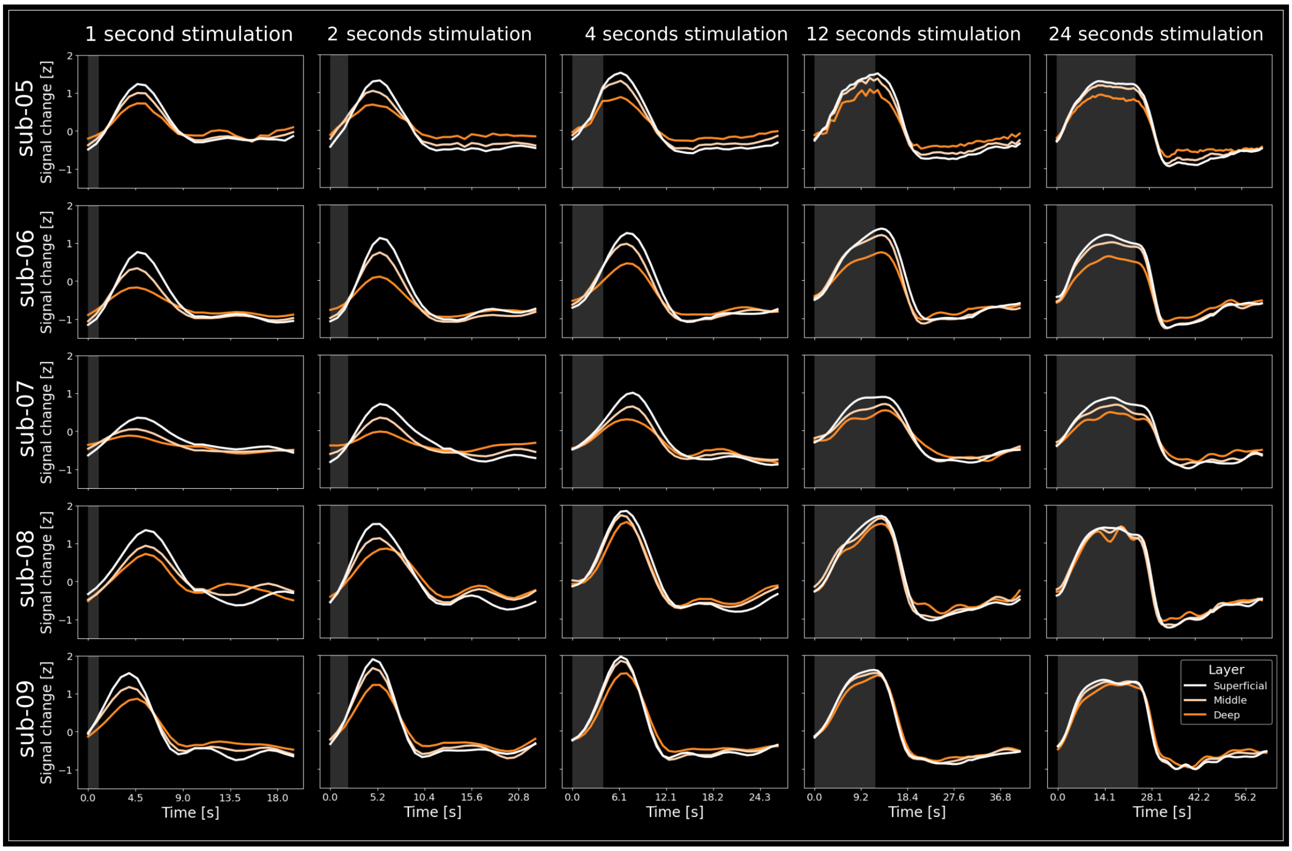

Figure S10: Z-scored BOLD results of individual participants. Same as **Figure S7** but with z-scored signal changes.

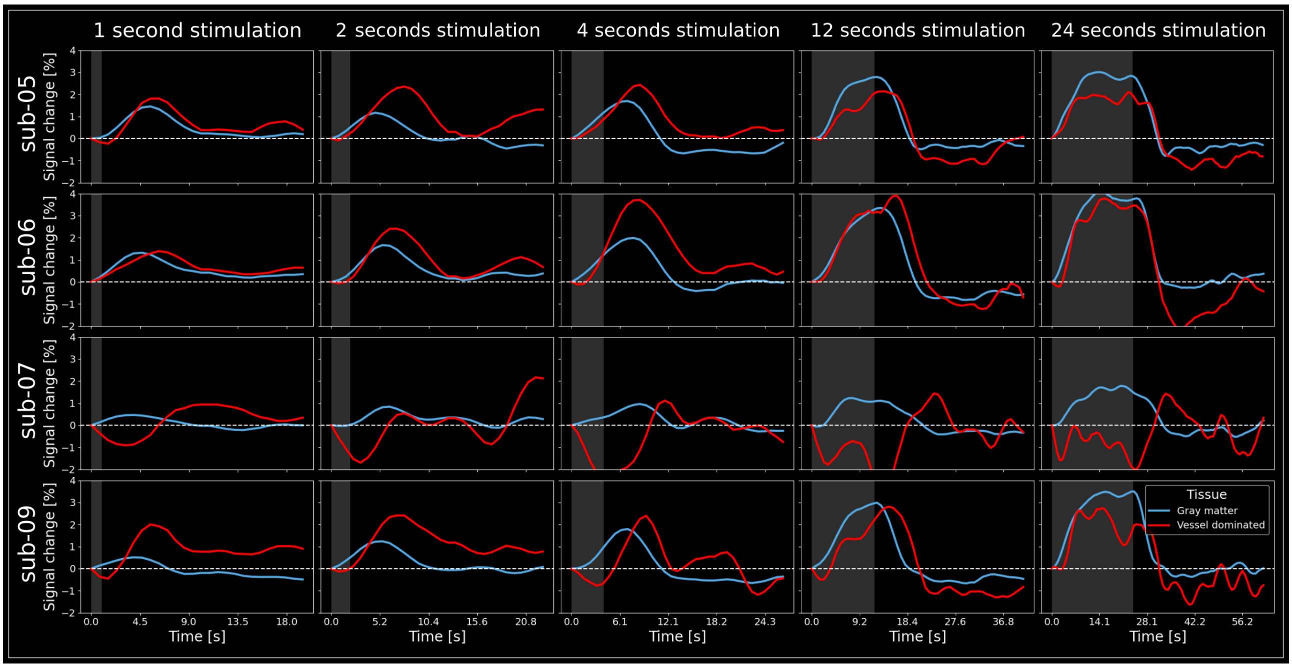

Figure S11: VASO responses in vessel-dominated and gray matter voxels of individual participants. Supplement to **Figure 6**

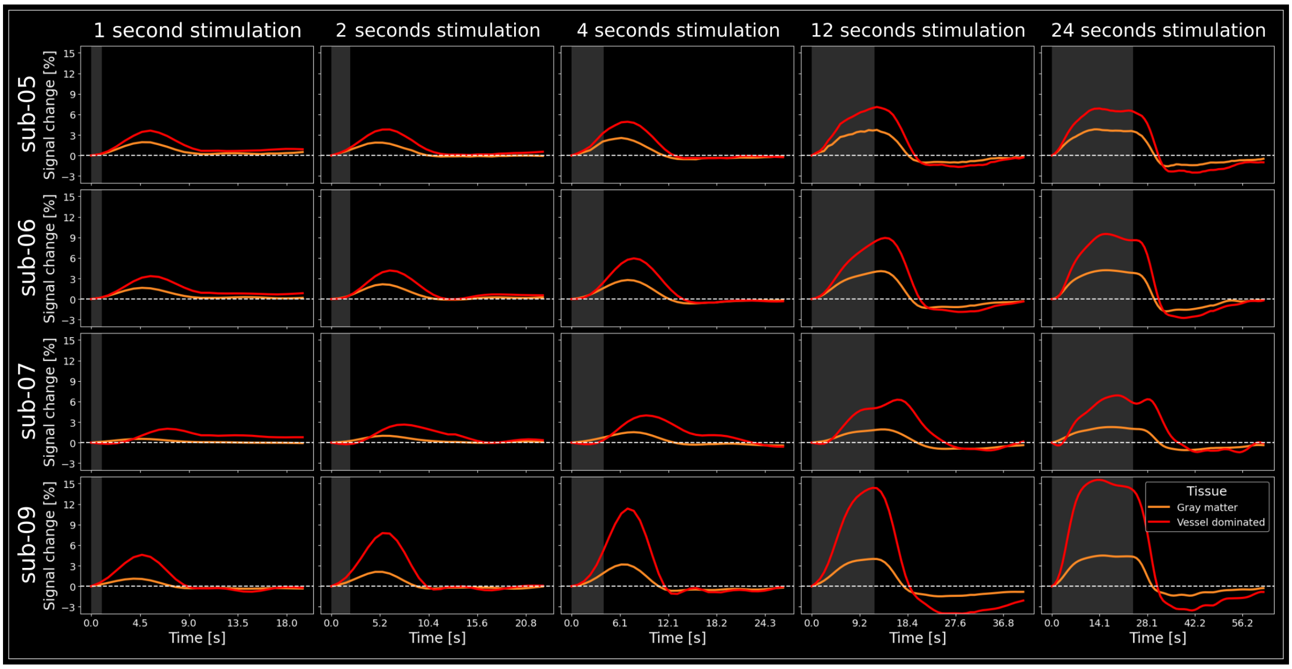

Figure S12: VASO responses in vessel-dominated and gray matter voxels of individual participants. Supplement to **Figure 6**

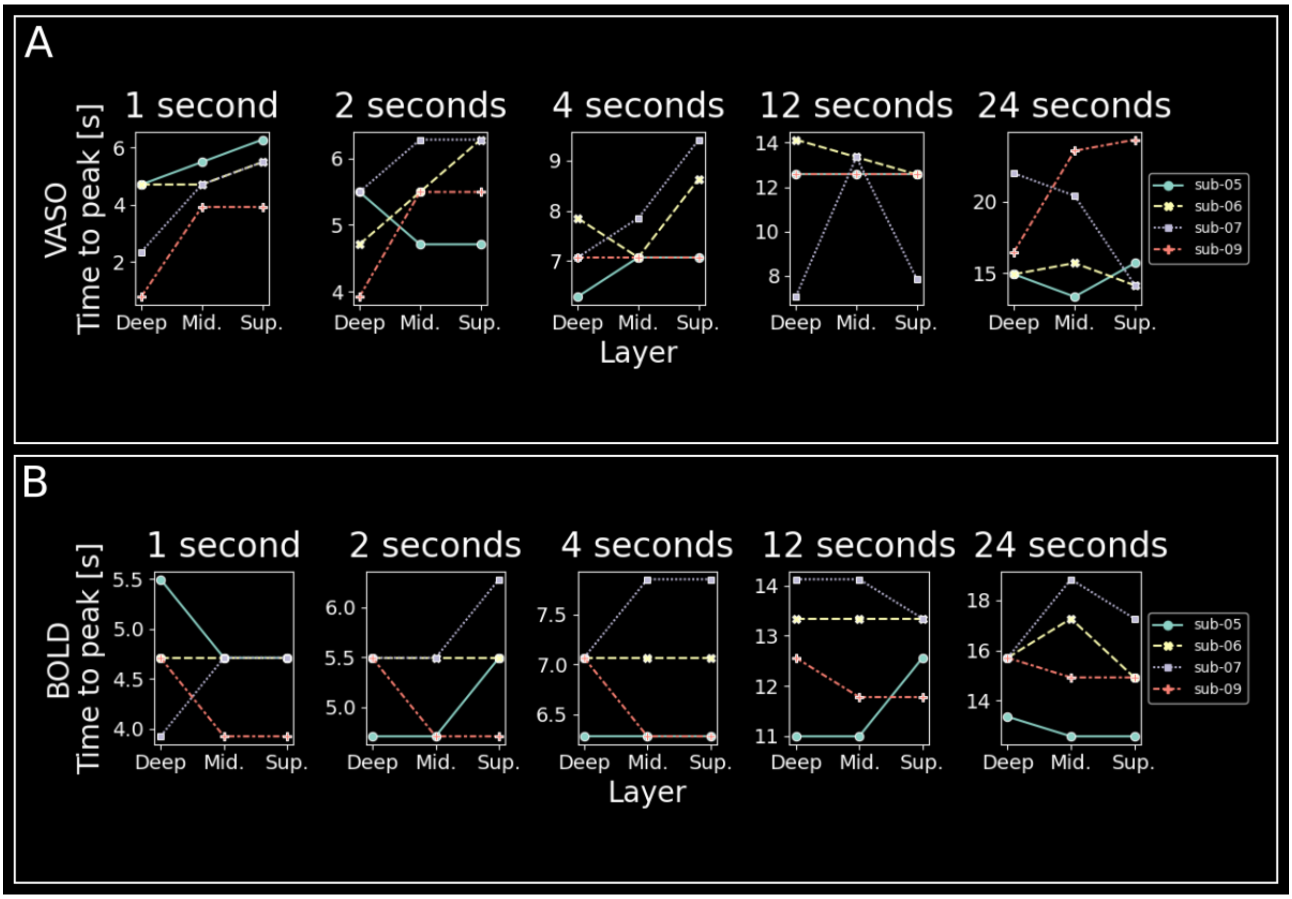

Figure S13: VASO and BOLD TTPs across stimulus durations in individual participants. A) Supplement to **Figure 5**
, showing TPPs of individual participants (indicated by different line and marker styles) for VASO across stimulus durations. The increase in TTP from deep to superficial layers for short stimuli is mostly consistent across participants. On the other hand, the longer TTP of middle compared to deep and superficial layers for 12 and 24 second stimulation (as observed on the group level) is driven by individual participants only. B) Same as A, but for BOLD data. Also here, the longer TTP of middle compared to deep and superficial layers for 24 second stimulation is driven by individual participants only.

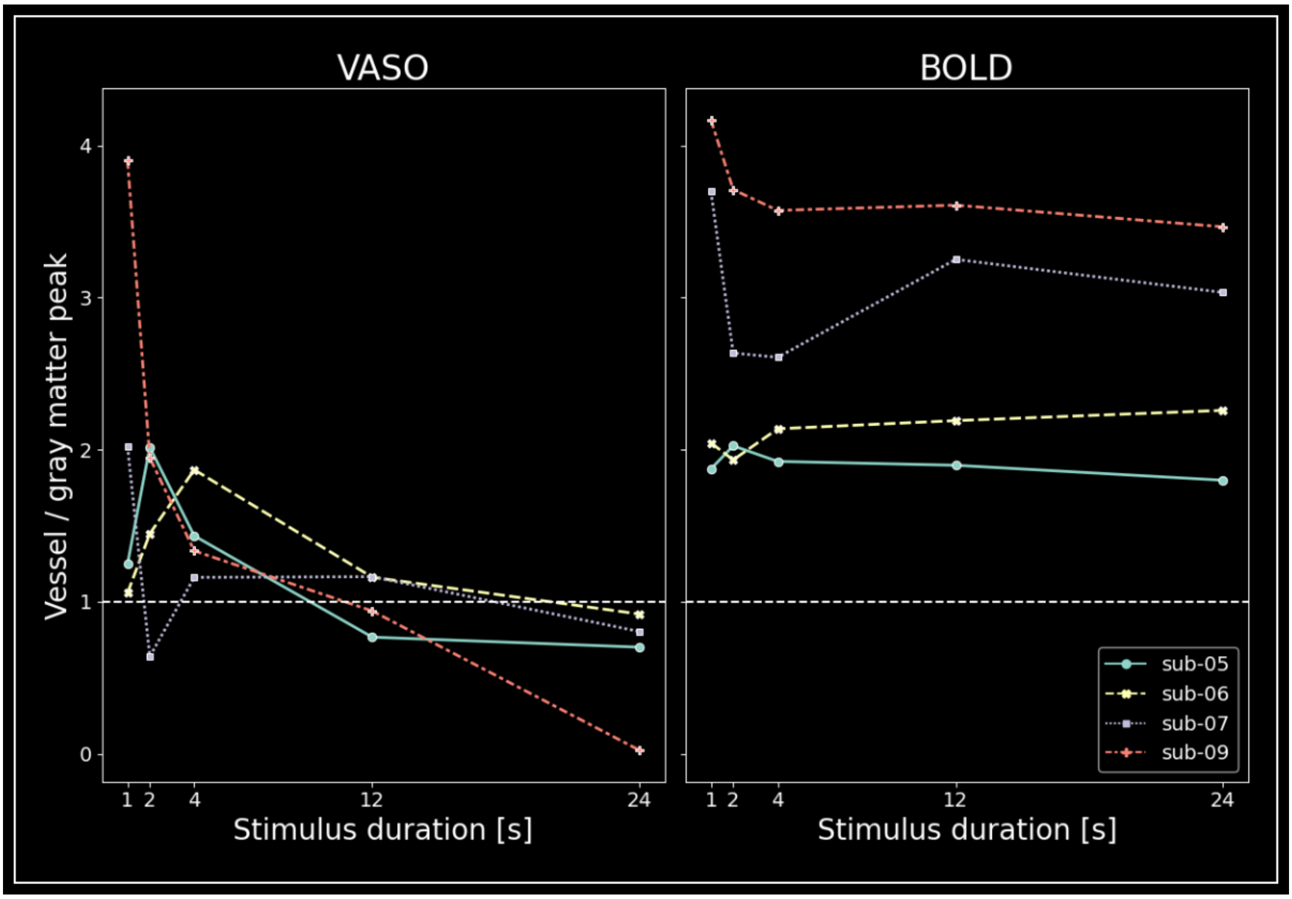

Figure S14: Ratio of vessel-dominated over gray matter peak signals for VASO and BOLD across stimulus durations in individual participants. Supplement to **Figure 7** , showing data from individual participants.

BrainVoyager pipeline for surface reconstruction

We applied the following BrainVoyager pipeline to generate inflated cortical surfaces for all participants. A video description can be found here: <https://www.youtube.com/watch?v=5Ik71Y_cLS8&ab_

1. MP2RAGE denoising
2. Brain extraction (mask size: -10)
3. Mask smoothing (FWHM 10 mm gaussian)
4. Erode mask (2 steps)
5. Intensity inhomogeneity correction
6. Iso voxeling to 0.5 mm isotropic resolution
7. ACPC transformation
8. Set Talairach box without interpolation
9. Label ventricles as WM (using talairach definition)
10. Remove cerebellum (using talairach definition)
11. Tissue contrast enhancement using sigma filter
12. Calculate gradients file
13. Adaptive wm-gm segmentation
14. Polish
15. Estimate GM (not crucial)
16. Disconnect hemispheres
17. Run bridge removal tool (for details, see Kriegeskorte & Goebel, 2001)
18. Reconstruct surface mesh
19. Mesh smoothing
20. Mesh simplification (80k vertices)
